# Supplementary figures and images for: Urban Wildlife Crisis: Australian Silver Gull Is a Bystander Host to Widespread Clinical Antibiotic Resistance
Source: mSystems. 2022 Apr 26;7(3):e00158-22. doi: 10.1128/msystems.00158-22 (PMC9238384; doi:10.1128/msystems.00158-22)

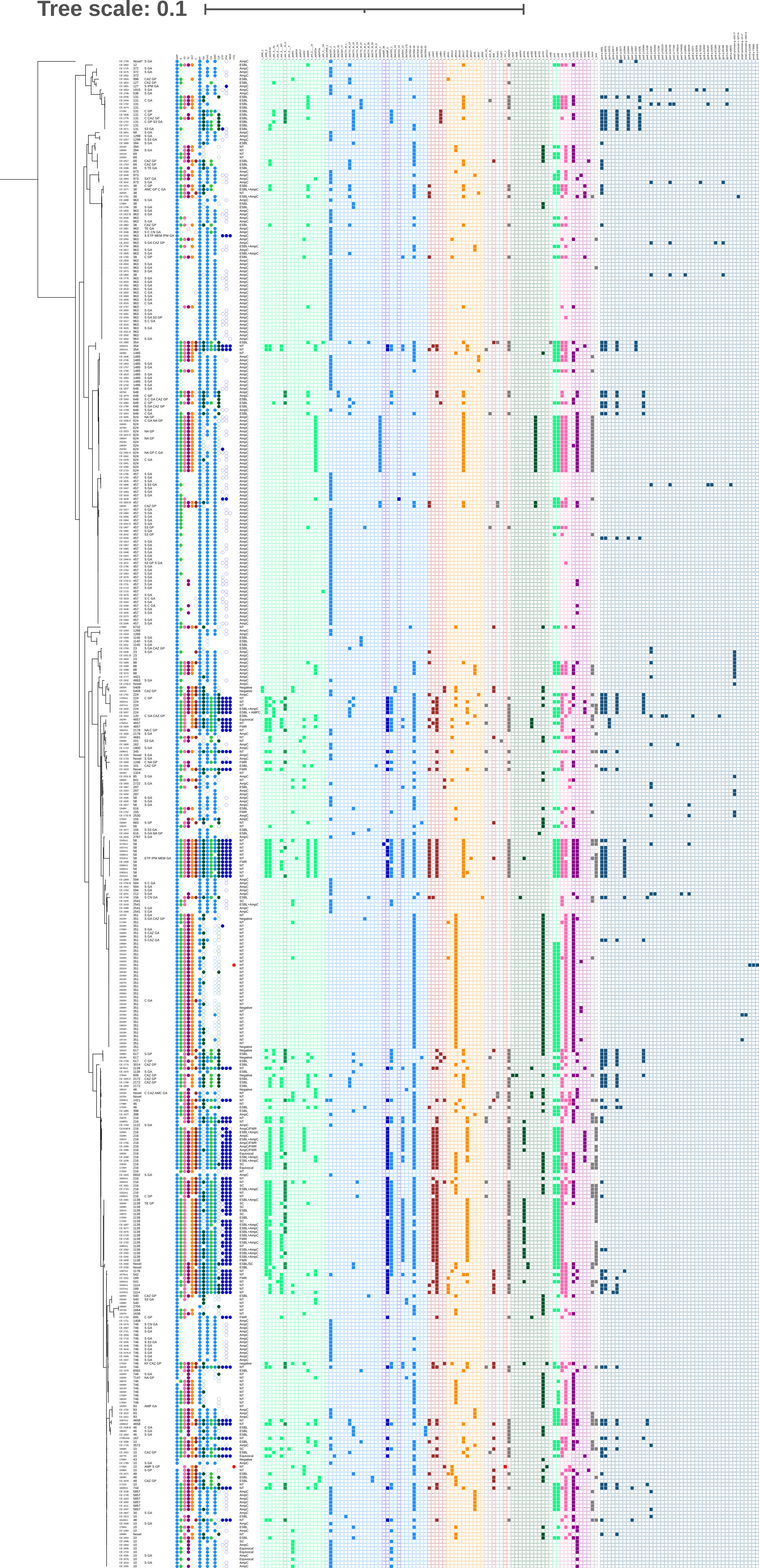

Supplement: FIG S1 [file msystems.00158-22-s0001.tif]
